# Supplementary material for: MonARCh: an actor based architecture for dynamic linked data monitoring
Source: PeerJ Comput Sci. 2024 Jul 12;10:e2133. doi: 10.7717/peerj-cs.2133 (PMC11323115; doi:10.7717/peerj-cs.2133)
Supplement: Supplemental Information 1 [file peerj-cs-10-2133-s001.pdf]

# APPENDICES

## A Evaluation Data and Query Templates

```
@prefix dbpr:   <http://dbpedia.org/resource/>
@prefix dbpo:   <http://dbpedia.org/ontology/>
@prefix rdf:    <http://www.w3.org/1999/02/22-rdf-syntax-ns#>
@prefix rdfs:   <http://www.w3.org/2000/01/rdf-schema#>
@prefix owl :   <http://www.w3.org/2002/07/owl#>
@prefix xsd :   <http://www.w3.org/2001/XMLSchema#>
@prefix nytr:   <http://data.nytimes.com/>
@prefix nyto:   <http://data.nytimes.com/elements/>
@prefix wsjr:   <https://www.wsj.com/market-data/quotes/>
@prefix stock:  <http://stockmarket.com/elements/>
-------------------- DBpedia -----------------------
dbr:exDbComp
  a                             dbpo:Company;
  rdfs:label                    "Ex␣Dbpedia␣Comp"^^xsd:string;
  dbpo:numberOfStaff            "1234"^^xsd:int;
  owl:sameAs                    nytr:exNyTComp;
  dbpo:industry                 dbpr:Electronics.
----------------------------------------------------
-------------------- NyTimes -----------------------
nytr:exNyTComp
  a                             nyto:Company;
  rdfs:label                    "Ex␣NyTimes␣Comp";
  nyto:associated_article_count "4321"^^xsd:int;
  nyto:reputation               "High"^^xsd:string;
  owl:sameAs                    wsjr:exWsjComp.
----------------------------------------------------
------------------- Stockmarket --------------------
nytr:exNyTComp
  a                             stock:Company;
  stock:market                  "NYSE"^^xsd:string;
  stock:currency                "USD"^^xsd:string;
  stock:stockPrice              "345"^^xsd:float;
  stock:companyName             "Ex␣Stock␣Comp"^^xsd:string;
  stock:trades                  "100"^^xsd:int;
  stock:shares                  "50"^^xsd:int;
  stock:valueChange             "24000"^^xsd:float;
  stock:changePercent           "7"^^xsd:float.
----------------------------------------------------
```

**Listing A1.** Example Company Data from the Evaluation Datasets in Rdf-Turtle Format

We have given a brief description about the evaluation data in Listing-A1. As to elaborate firstly, DBpedia has descriptive information about companies with the properties of "label", "type", "number of staff", and "industry". For the "number of employees" property, we generated a random value from 1-10000 to 90000-100000 for each of the 500 companies. There are ten distinct ranges, each with 500 companies, for a total of 5000 companies with this property value. There are six industry property value ranges held by the first 500 and last 100 companies. These "industry" values are "Bank", "Airline", "Software", "Electronics", "Health" and "Restaurant", and each value is held by 100 companies, for a total of 600.

Second, NyTimes has news and media information, as well as company-related updates. "Associated article count" and "reputation" are two important properties. The "Associated article count" was initially set to 0 and was updated by the NyTimes Updater after each time period. Every 1000 companies have one of the reputation values "Very High", "High", "Medium", "Low" or "Very Low" for a total of 5000 companies. Furthermore, the first 500 and last 100 companies have secondary reputations as "Elite" and "Initial" respectively. There is also a linkset with the "same as" property that connects the New York Times to the Wall Street Journal, which is a virtual dataset. This linkset was created to improve query diversity in the NyTimes dataset. NyTimes also has some descriptive properties like "type" and "label".

Finally, the Stockmarket dataset has financial information and updates about companies alongside some descriptive properties such as "type" and "company name". Every 1000 companies have the same (market-currency) pair between "NYSE"-"USD", "TSE"-"JPY", "FWB"-"EUR", "LSE"-"GBP" and "BST"-"TRY" totalling 5000 companies. Special "value change" and "change percent" rates apply to the

first 500 and last 100 companies. There are six different ranges of these values, and each range is shared by 100 companies, for a total of 600. For each company, a "stock price" was generated at random from 1 to 500 and was updated on a regular basis by Stockmarket Updater. Values for "trades" were generated based on "stock price" and values for "shares" were generated based on "trades" by multiplying by a random value ranging from 10 to 250. These two values were used to supplement the virtual data in the Stockmarket dataset.

```
PREFIX rdf: <http://www.w3.org/1999/02/22-rdf-syntax-ns#>
PREFIX rdfs: <http://www.w3.org/2000/01/rdf-schema#>
PREFIX dbpo: <http://dbpedia.org/ontology/>
PREFIX nytimes: <http://data.nytimes.com/elements/>
PREFIX stockmarket: <http://stockmarket.com/elements/>
PREFIX owl: <http://www.w3.org/2002/07/owl#>
Select * where {
{BIND(<http://155.223.25.4:8890/dbpedia/sparql> AS ?ser)}
UNION
{BIND(<http://155.223.25.1:8890/nytimes/sparql> AS ?ser)}
Service ?ser {
   ?company rdf:type ?type.
   VALUES (?type) {
      (dbpo:Company)(nytimes:Company)(stockmarket:Company)
   }
   ?company rdfs:label ?label.
   ?company owl:sameAs ?sameCompany.
 }
service <http://155.223.25.2:8890/stockmarket/sparql> {
   ?company stockmarket:market ?market.
   ?company stockmarket:currency ?currency.
   ?company stockmarket:stockPrice ?stockPrice.
 }
}
```

**Listing A2.** Least Selective Query

```
PREFIX rdf: <http://www.w3.org/1999/02/22-rdf-syntax-ns#>
PREFIX rdfs: <http://www.w3.org/2000/01/rdf-schema#>
PREFIX xsd: <http://www.w3.org/2001/XMLSchema#>
PREFIX dbo: <http://dbpedia.org/ontology/>
PREFIX dbpedia: <http://dbpedia.org/resource/>
PREFIX nytimes: <http://data.nytimes.com/elements/>
PREFIX stockmarket: <http://stockmarket.com/elements/>
PREFIX owl: <http://www.w3.org/2002/07/owl#>
Select * where {
service <http://155.223.25.4:8890/dbpedia/sparql> {
   ?dbpediaCompany rdf:type dbo:Company.
   ?dbpediaCompany dbo:numberOfStaff ?staffCount.
   ?dbpediaCompany owl:sameAs ?nytCompany.
   FILTER (?staffCount>lowerBound && ?staffCount<=upperBound)
  }
service <http://155.223.25.1:8890/nytimes/sparql> {
   ?nytCompany rdf:type nytimes:Company.
   ?nytCompany nytimes:reputation ?reputation.
   ?nytCompany nytimes:associated_article_count ?articleCount.
   VALUES (?reputation) {
      ('Very␣High'^^xsd:string)('High'^^xsd:string)(...)
    }
  }
service <http://155.223.25.2:8890/stockmarket/sparql> {
   ?nytCompany rdf:type stockmarket:Company.
   ?nytCompany stockmarket:market ?market.
   ?nytCompany stockmarket:currency ?currency.
   ?nytCompany stockmarket:stockPrice ?stockPrice.
   VALUES (?market){
      ('NYSE'^^xsd:string)('TSE'^^xsd:string)(...)
    }
  }
}
```

**Listing A3.** Low Selective Query

```
PREFIX rdf: <http://www.w3.org/1999/02/22-rdf-syntax-ns#>
PREFIX rdfs: <http://www.w3.org/2000/01/rdf-schema#>
PREFIX xsd: <http://www.w3.org/2001/XMLSchema#>
PREFIX dbo: <http://dbpedia.org/ontology/>
PREFIX dbpedia: <http://dbpedia.org/resource/>
PREFIX nytimes: <http://data.nytimes.com/elements/>
PREFIX stockmarket: <http://stockmarket.com/elements/>
PREFIX owl: <http://www.w3.org/2002/07/owl#>
Select * where {
service <http://155.223.25.4:8890/dbpedia/sparql> {
   dbpedia:exComp dbo:numberOfStaff ?nos.
   dbpedia:exComp owl:sameAs ?nytCompany.
 }
service <http://155.223.25.1:8890/nytimes/sparql> {
   ?nytCompany rdf:type nytimes:Company.
   ?nytCompany nytimes:reputation ?reputation.
   ?nytCompany nytimes:associated_article_count ?articleCount.
   VALUES (?reputation) {
     ('Very␣High'^^xsd:string)('High'^^xsd:string)(...)
   }
 }
service <http://155.223.25.2:8890/stockmarket/sparql> {
   ?nytCompany rdf:type stockmarket:Company.
   ?nytCompany stockmarket:market ?market.
   ?nytCompany stockmarket:currency ?currency.
   ?nytCompany stockmarket:stockPrice ?stockPrice.
   VALUES (?market) {
     ('NYSE'^^xsd:string)('TSE'^^xsd:string)(...)
   }
 }
}
```

**Listing A4.** Middle Selective Query

```
PREFIX rdf: <http://www.w3.org/1999/02/22-rdf-syntax-ns#>
PREFIX rdfs: <http://www.w3.org/2000/01/rdf-schema#>
PREFIX xsd: <http://www.w3.org/2001/XMLSchema#>
PREFIX dbo: <http://dbpedia.org/ontology/>
PREFIX dbpedia: <http://dbpedia.org/resource/>
PREFIX nytimes: <http://data.nytimes.com/elements/>
PREFIX stockmarket: <http://stockmarket.com/elements/>
PREFIX owl: <http://www.w3.org/2002/07/owl#>
Select * where {
service <http://155.223.25.4:8890/dbpedia/sparql> {
   VALUES (?industry) {
      (dbpedia:Bank)(dbpedia:Airline)(...)
   }
   ?dbpediaCompany rdf:type dbo:Company.
   ?dbpediaCompany dbo:numberOfStaff ?staffCount.
   ?dbpediaCompany dbo:industry ?industry.
   ?dbpediaCompany owl:sameAs ?nytCompany.
   FILTER (?staffCount>90000&&?staffCount<=100000)
 }
service <http://155.223.25.1:8890/nytimes/sparql> {
   ?nytCompany rdf:type nytimes:Company.
   ?nytCompany nytimes:reputation 'Elite'^^xsd:string.
   ?nytCompany nytimes:associated_article_count ?articleCount.
 }
service <http://155.223.25.2:8890/stockmarket/sparql> {
   ?nytCompany rdf:type stockmarket:Company.
   ?nytCompany stockmarket:market ?market.
   ?nytCompany stockmarket:currency ?currency.
   ?nytCompany stockmarket:stockPrice ?stockPrice.
   ?nytCompany stockmarket:valueChange ?valueChange.
   FILTER (?valueChange>=lowerBound&&?valueChange<upperBound)
 }
}
```

**Listing A5.** High Selective Query

```
PREFIX rdf: <http://www.w3.org/1999/02/22-rdf-syntax-ns#>
PREFIX rdfs: <http://www.w3.org/2000/01/rdf-schema#>
PREFIX xsd: <http://www.w3.org/2001/XMLSchema#>
PREFIX dbo: <http://dbpedia.org/ontology/>
PREFIX dbpedia: <http://dbpedia.org/resource/>
PREFIX nytimes: <http://data.nytimes.com/elements/>
PREFIX stockmarket: <http://stockmarket.com/elements/>
PREFIX owl: <http://www.w3.org/2002/07/owl#>
Select * where {
service <http://155.223.25.4:8890/dbpedia/sparql> {
   dbpedia:exComp dbo:numberOfStaff ?nos.
   dbpedia:exComp owl:sameAs ?nytCompany.
 }
service <http://155.223.25.1:8890/nytimes/sparql> {
   ?nytCompany rdf:type nytimes:Company.
   ?nytCompany nytimes:reputation 'Elite'^^xsd:string.
   ?nytCompany nytimes:associated_article_count ?articleCount.
 }
service <http://155.223.25.2:8890/stockmarket/sparql> {
   ?nytCompany rdf:type stockmarket:Company.
   ?nytCompany stockmarket:market ?market.
   ?nytCompany stockmarket:currency ?currency.
   ?nytCompany stockmarket:stockPrice ?stockPrice.
   ?nytCompany stockmarket:valueChange ?valueChange.
   FILTER (?valueChange>=lowerBound&&?valueChange<upperBound)
 }
}
```

**Listing A6.** Most Selective Query

## B Query and Monitoring Performance Tables

**Table B1.** Query and Monitoring Performance Results of the Least Selective Query

| SC Result Filtering (%) | Number of Monitored Queries | Avg Query Processing Time (s) | | Max Query Processing Time (s) | | Avg Change Notification Time (s) | | Max Change Notification Time (s) | |
|---|---|---|---|---|---|---|---|---|---|
| | | Node-1 | Node-2 | Node-1 | Node-2 | Node-1 | Node-2 | Node-1 | Node-2 |
| - | 100 | 6.42 | 7.25 | 11.89 | 12.18 | 2.49 | 2.48 | 8.63 | 8.98 |
| - | 200 | 16.36 | 14.23 | 47.04 | 29.47 | 3.22 | 3.23 | 14.46 | 15.32 |
| - | 300 | 25.07 | 27.35 | 96.01 | 95.80 | 4.09 | 4.38 | 19.17 | 17.02 |

**Table B2.** Query and Monitoring Performance Results of the Low Selective Query

| SC Result Filtering (%) | Number of Monitored Queries | Avg Query Processing Time (s) | | Max Query Processing Time (s) | | Avg Change Notification Time (s) | | Max Change Notification Time (s) | |
|---|---|---|---|---|---|---|---|---|---|
| | | Node-1 | Node-2 | Node-1 | Node-2 | Node-1 | Node-2 | Node-1 | Node-2 |
| 0 | 100 | 6.90 | 7.20 | 18.54 | 18.14 | 3.17 | 3.21 | 9.54 | 9.01 |
| 0 | 200 | 13.19 | 14.21 | 25.21 | 27.81 | 6.22 | 7.12 | 16.38 | 16.22 |
| 0 | 300 | 23.23 | 20.98 | 47.46 | 45.86 | 11.98 | 8.00 | 251.80 | 265.18 |
| 20 | 100 | 5.80 | 5.62 | 11.49 | 11.87 | 2.84 | 2.41 | 9.68 | 7.02 |
| 20 | 200 | 10.12 | 8.27 | 20.80 | 19.54 | 4.99 | 4.72 | 12.29 | 11.68 |
| 20 | 300 | 15.29 | 15.83 | 25.57 | 30.39 | 6.33 | 6.40 | 17.75 | 18.28 |
| 20 | 400 | 22.49 | 23.53 | 55.14 | 54.84 | 9.94 | 5.47 | 86.79 | 16.61 |
| 40 | 100 | 3.81 | 4.24 | 6.40 | 8.72 | 1.87 | 2.00 | 5.56 | 5.39 |
| 40 | 200 | 6.63 | 6.57 | 20.97 | 21.21 | 3.24 | 3.26 | 11.75 | 9.64 |
| 40 | 300 | 8.90 | 9.63 | 15.11 | 18.17 | 4.15 | 5.05 | 17.42 | 12.65 |
| 40 | 400 | 12.89 | 14.29 | 33.47 | 33.25 | 4.90 | 5.50 | 16.01 | 17.13 |
| 40 | 500 | 15.78 | 18.24 | 32.94 | 34.58 | 5.76 | 5.85 | 83.88 | 28.52 |
| 60 | 100 | 2.12 | 2.15 | 3.64 | 3.80 | 1.36 | 1.34 | 3.76 | 3.52 |
| 60 | 200 | 4.60 | 4.49 | 8.50 | 8.23 | 2.05 | 2.00 | 6.54 | 6.45 |
| 60 | 300 | 5.76 | 5.68 | 13.61 | 13.80 | 2.55 | 2.77 | 11.04 | 11.30 |
| 60 | 400 | 8.03 | 8.17 | 17.24 | 16.61 | 3.00 | 4.02 | 10.67 | 12.98 |
| 60 | 500 | 10.75 | 10.83 | 19.17 | 18.14 | 3.68 | 4.89 | 20.16 | 18.50 |
| 60 | 750 | 16.87 | 16.79 | 36.47 | 36.59 | 8.37 | 4.54 | 51.16 | 47.56 |
| 80 | 500 | 4.58 | 4.18 | 8.35 | 7.72 | 2.23 | 2.00 | 7.15 | 6.90 |
| 80 | 750 | 7.72 | 7.28 | 18.23 | 18.01 | 3.03 | 3.18 | 14.34 | 12.80 |
| 80 | 1000 | 8.73 | 8.29 | 17.96 | 17.34 | 4.17 | 3.58 | 18.93 | 18.01 |
| 80 | 1250 | 12.35 | 12.93 | 26.96 | 27.92 | 3.75 | 4.22 | 20.71 | 20.39 |
| 80 | 1500 | 20.65 | 21.09 | 56.57 | 58.42 | 3.92 | 6.79 | 47.00 | 135.41 |

**Table B3.** Query and Monitoring Performance Results of the Middle Selective Query

| SC Result Filtering (%) | Number of Monitored Queries | Avg Query Processing Time (s) | | Max Query Processing Time (s) | | Avg Change Notification Time (s) | | Max Change Notification Time (s) | |
|---|---|---|---|---|---|---|---|---|---|
| | | **Node-1** | **Node-2** | **Node-1** | **Node-2** | **Node-1** | **Node-2** | **Node-1** | **Node-2** |
| 0 | 100 | 33.82 | 29.54 | 46.26 | 44.73 | 8.85 | 9.36 | 31.00 | 26.06 |
| 0 | 200 | 70.85 | 69.65 | 97.57 | 97.41 | 14.16 | 14.38 | 47.11 | 60.94 |
| 0 | 300 | 22.13 | 20.68 | 46.55 | 47.30 | 8.60 | 9.80 | 31.52 | 27.96 |
| 0 | 400 | 41.02 | 37.09 | 96.80 | 74.46 | 8.01 | 9.50 | 36.47 | 34.36 |
| 0 | 500 | 50.58 | 51.61 | 105.19 | 110.25 | 15.88 | 16.00 | 69.83 | 64.19 |
| 20 | 100 | 11.14 | 11.79 | 16.91 | 16.49 | 4.26 | 5.93 | 11.15 | 13.63 |
| 20 | 200 | 13.96 | 15.75 | 22.86 | 30.04 | 4.76 | 5.73 | 19.32 | 17.24 |
| 20 | 300 | 18.91 | 18.72 | 32.22 | 31.91 | 5.30 | 8.16 | 21.67 | 23.86 |
| 20 | 400 | 29.27 | 26.91 | 62.65 | 58.80 | 7.24 | 10.11 | 25.00 | 28.01 |
| 20 | 500 | 27.64 | 31.80 | 49.28 | 55.64 | 9.36 | 10.84 | 38.34 | 40.37 |
| 20 | 750 | 43.18 | 43.63 | 195.37 | 193.27 | 4.60 | 4.79 | 38.50 | 15.03 |
| 40 | 100 | 9.95 | 8.11 | 15.23 | 13.74 | 3.31 | 3.03 | 10.69 | 9.96 |
| 40 | 200 | 9.60 | 9.22 | 16.38 | 16.79 | 3.64 | 3.87 | 12.48 | 12.13 |
| 40 | 300 | 14.04 | 13.55 | 24.46 | 24.36 | 3.76 | 4.31 | 16.99 | 16.50 |
| 40 | 400 | 17.71 | 15.12 | 30.41 | 26.13 | 4.92 | 4.30 | 18.42 | 18.31 |
| 40 | 500 | 19.91 | 17.26 | 45.80 | 38.05 | 6.49 | 6.33 | 22.96 | 24.07 |
| 40 | 750 | 32.42 | 32.22 | 67.91 | 64.17 | 8.35 | 9.82 | 32.82 | 39.67 |
| 40 | 1000 | 25.68 | 26.11 | 64.37 | 61.53 | 19.15 | 8.32 | 275.83 | 45.92 |
| 60 | 250 | 16.22 | 14.62 | 21.73 | 21.16 | 3.46 | 3.14 | 14.61 | 14.59 |
| 60 | 500 | 13.84 | 14.18 | 25.98 | 25.59 | 2.97 | 4.00 | 18.44 | 17.73 |
| 60 | 750 | 18.11 | 20.91 | 36.13 | 35.08 | 5.55 | 7.32 | 21.11 | 21.99 |
| 60 | 1000 | 16.37 | 16.54 | 46.67 | 46.57 | 4.14 | 5.67 | 17.89 | 17.99 |
| 60 | 1250 | 19.55 | 19.02 | 64.47 | 63.69 | 5.12 | 6.87 | 38.54 | 41.63 |
| 80 | 500 | 28.56 | 29.10 | 40.53 | 40.83 | 1.81 | 1.80 | 9.96 | 9.52 |
| 80 | 750 | 45.83 | 44.41 | 65.85 | 65.96 | 2.32 | 2.52 | 14.06 | 12.69 |
| 80 | 1000 | 25.33 | 26.89 | 41.69 | 41.93 | 4.21 | 6.34 | 20.53 | 28.74 |
| 80 | 1500 | 62.25 | 63.20 | 152.49 | 152.14 | 5.02 | 11.93 | 34.41 | 35.42 |
| 80 | 2000 | 14.20 | 18.26 | 32.17 | 42.01 | 3.89 | 7.31 | 24.62 | 25.88 |
| 80 | 2500 | 30.32 | 23.68 | 139.20 | 115.68 | 5.19 | 6.43 | 71.43 | 64.42 |

**Table B4.** Query and Monitoring Performance Results of the High Selective Query

| SC Result Filtering (%) | Number of Monitored Queries | Avg Query Processing Time (s) | | Max Query Processing Time (s) | | Avg Change Notification Time (s) | | Max Change Notification Time (s) | |
|---|---|---|---|---|---|---|---|---|---|
| | | **Node-1** | **Node-2** | **Node-1** | **Node-2** | **Node-1** | **Node-2** | **Node-1** | **Node-2** |
| 0 | 500 | 24.63 | 24.41 | 33.36 | 33.13 | 2.86 | 1.79 | 13.69 | 8.63 |
| 0 | 1000 | 26.71 | 23.28 | 43.77 | 35.40 | 4.19 | 3.17 | 34.57 | 16.38 |
| 0 | 1500 | 16.26 | 19.46 | 36.35 | 36.74 | 3.87 | 4.82 | 20.73 | 19.29 |
| 0 | 2000 | 18.11 | 23.54 | 54.43 | 56.76 | 5.22 | 5.92 | 29.95 | 25.54 |
| 0 | 2500 | 12.92 | 15.53 | 30.63 | 45.25 | 3.17 | 5.58 | 18.10 | 28.34 |
| 20 | 500 | 17.67 | 18.70 | 28.82 | 28.99 | 1.25 | 1.96 | 8.50 | 9.15 |
| 20 | 1000 | 17.11 | 17.28 | 26.39 | 26.13 | 2.83 | 4.13 | 20.05 | 21.25 |
| 20 | 1500 | 20.49 | 21.18 | 39.42 | 40.69 | 4.40 | 6.77 | 31.02 | 25.59 |
| 20 | 2000 | 23.46 | 25.19 | 46.75 | 48.09 | 3.41 | 7.89 | 18.90 | 29.81 |
| 20 | 2500 | 22.94 | 24.28 | 48.37 | 48.80 | 4.65 | 6.65 | 34.63 | 34.74 |
| 20 | 3000 | 9.95 | 10.98 | 21.64 | 22.06 | 3.67 | 5.83 | 24.96 | 21.51 |
| 40 | 500 | 13.62 | 12.22 | 23.27 | 23.24 | 1.67 | 0.97 | 11.84 | 7.25 |
| 40 | 1000 | 12.52 | 12.36 | 23.07 | 22.52 | 1.99 | 3.82 | 14.04 | 16.66 |
| 40 | 2000 | 37.64 | 33.68 | 68.08 | 56.06 | 5.24 | 7.77 | 32.67 | 30.51 |
| 40 | 3000 | 25.82 | 27.46 | 64.56 | 65.02 | 4.43 | 6.90 | 28.50 | 26.57 |
| 40 | 4000 | 89.49 | 64.00 | 221.18 | 158.22 | 5.96 | 8.47 | 73.28 | 41.28 |
| 40 | 4500 | 38.24 | 44.06 | 119.42 | 123.16 | 5.07 | 9.89 | 27.70 | 93.35 |
| 60 | 500 | 11.40 | 10.61 | 18.09 | 17.93 | 0.79 | 0.95 | 4.06 | 6.10 |
| 60 | 1000 | 18.95 | 20.33 | 37.82 | 37.39 | 1.34 | 1.40 | 9.65 | 8.97 |
| 60 | 2000 | 19.50 | 17.86 | 35.03 | 34.72 | 3.30 | 3.81 | 20.08 | 28.57 |
| 60 | 3000 | 23.62 | 24.02 | 58.71 | 64.14 | 5.19 | 7.10 | 34.86 | 36.82 |
| 60 | 4000 | 35.38 | 28.36 | 98.37 | 90.62 | 6.25 | 9.65 | 37.34 | 31.13 |
| 60 | 5000 | 30.41 | 31.28 | 85.20 | 89.71 | 5.24 | 7.23 | 32.66 | 31.36 |
| 60 | 6000 | 37.72 | 40.42 | 122.14 | 117.28 | 5.26 | 6.30 | 53.03 | 204.07 |
| 80 | 500 | 8.37 | 8.49 | 11.93 | 12.09 | 0.44 | 0.92 | 1.79 | 4.56 |
| 80 | 1000 | 5.74 | 5.79 | 12.55 | 12.06 | 1.45 | 1.43 | 8.64 | 8.58 |
| 80 | 2500 | 5.00 | 4.97 | 12.04 | 12.19 | 1.55 | 1.51 | 8.22 | 10.69 |
| 80 | 5000 | 4.90 | 4.95 | 12.00 | 11.70 | 1.96 | 1.75 | 11.61 | 10.07 |
| 80 | 7500 | 6.78 | 8.47 | 17.50 | 21.94 | 2.63 | 2.96 | 16.47 | 16.86 |
| 80 | 10000 | 9.75 | 9.39 | 21.94 | 21.40 | 3.73 | 3.87 | 24.28 | 21.37 |
| 80 | 12500 | 8.68 | 9.80 | 27.80 | 30.92 | 6.60 | 6.34 | 49.35 | 42.03 |

**Table B5.** Query and Monitoring Performance Results of the Most Selective Query

| SC Result Filtering (%) | Number of Monitored Queries | Avg Query Processing Time (s) | | Max Query Processing Time (s) | | Avg Change Notification Time (s) | | Max Change Notification Time (s) | |
|---|---|---|---|---|---|---|---|---|---|
| | | Node-1 | Node-2 | Node-1 | Node-2 | Node-1 | Node-2 | Node-1 | Node-2 |
| 0 | 500 | 13.70 | 14.38 | 23.03 | 22.95 | 0.44 | 0.52 | 3.32 | 3.01 |
| 0 | 1000 | 13.16 | 13.11 | 20.68 | 20.79 | 3.54 | 3.00 | 20.67 | 13.31 |
| 0 | 2000 | 5.64 | 5.71 | 12.13 | 12.29 | 1.82 | 2.51 | 10.50 | 11.57 |
| 0 | 3000 | 6.92 | 8.09 | 24.76 | 25.21 | 2.96 | 3.97 | 12.60 | 14.43 |
| 0 | 4000 | 10.29 | 11.59 | 22.94 | 22.97 | 2.92 | 4.67 | 18.58 | 19.41 |
| 0 | 5000 | 15.13 | 17.43 | 34.84 | 35.71 | 5.72 | 6.61 | 65.24 | 246.16 |
| 20 | 500 | 12.11 | 13.01 | 18.28 | 18.38 | 0.51 | 0.61 | 3.09 | 2.95 |
| 20 | 1000 | 24.08 | 22.38 | 37.03 | 36.41 | 1.57 | 1.78 | 9.00 | 10.72 |
| 20 | 2000 | 25.97 | 27.63 | 50.39 | 51.14 | 3.15 | 8.29 | 18.94 | 35.66 |
| 20 | 3000 | 30.38 | 29.72 | 72.52 | 71.09 | 6.27 | 7.56 | 34.45 | 29.56 |
| 20 | 4000 | 29.61 | 30.74 | 72.39 | 74.93 | 4.69 | 6.85 | 30.68 | 39.69 |
| 20 | 5000 | 34.04 | 33.53 | 74.86 | 83.51 | 5.68 | 6.20 | 42.75 | 35.93 |
| 20 | 6000 | 25.93 | 28.39 | 64.02 | 65.91 | 4.31 | 6.89 | 66.93 | 48.10 |
| 40 | 500 | 10.80 | 11.99 | 17.03 | 16.35 | 0.98 | 1.44 | 7.45 | 7.90 |
| 40 | 1000 | 16.64 | 17.22 | 30.09 | 29.81 | 1.16 | 0.87 | 8.24 | 5.44 |
| 40 | 2000 | 17.13 | 17.39 | 29.45 | 29.10 | 3.78 | 5.60 | 24.97 | 25.56 |
| 40 | 3000 | 13.92 | 14.61 | 33.06 | 33.31 | 3.98 | 4.07 | 18.24 | 20.50 |
| 40 | 4000 | 20.25 | 21.75 | 53.19 | 55.35 | 3.61 | 5.61 | 33.01 | 31.89 |
| 40 | 5000 | 22.12 | 21.26 | 57.84 | 58.67 | 5.14 | 7.34 | 27.29 | 27.05 |
| 40 | 6000 | 14.81 | 14.23 | 38.34 | 37.36 | 3.09 | 4.37 | 22.04 | 22.80 |
| 40 | 7000 | 18.48 | 19.97 | 52.89 | 52.79 | 4.53 | 6.41 | 43.58 | 48.39 |
| 60 | 500 | 8.63 | 8.26 | 12.56 | 12.22 | 0.44 | 0.40 | 3.32 | 2.97 |
| 60 | 1000 | 12.46 | 13.96 | 21.54 | 22.31 | 1.39 | 1.56 | 6.98 | 9.19 |
| 60 | 2000 | 11.95 | 12.02 | 24.86 | 24.61 | 4.86 | 3.38 | 20.52 | 19.42 |
| 60 | 3000 | 18.22 | 14.97 | 37.34 | 26.20 | 3.48 | 5.75 | 23.36 | 24.43 |
| 60 | 4000 | 6.79 | 6.45 | 17.92 | 18.42 | 2.34 | 2.80 | 13.82 | 12.69 |
| 60 | 5000 | 6.74 | 6.96 | 15.48 | 14.69 | 2.88 | 2.85 | 16.90 | 20.54 |
| 60 | 6000 | 9.50 | 9.01 | 24.03 | 23.10 | 3.19 | 4.21 | 20.89 | 21.51 |
| 60 | 7000 | 10.52 | 9.66 | 27.28 | 26.28 | 3.48 | 3.28 | 25.66 | 23.80 |
| 60 | 8000 | 12.04 | 12.29 | 41.09 | 37.62 | 4.73 | 5.13 | 28.24 | 25.91 |
| 60 | 9000 | 17.91 | 15.38 | 41.48 | 39.70 | 5.72 | 6.28 | 71.70 | 380.94 |
| 80 | 500 | 6.29 | 7.16 | 9.26 | 10.10 | 0.29 | 0.56 | 1.21 | 3.20 |
| 80 | 1000 | 4.39 | 5.31 | 9.27 | 9.58 | 0.91 | 1.62 | 5.19 | 6.73 |
| 80 | 2500 | 7.10 | 7.19 | 14.79 | 15.37 | 2.30 | 3.09 | 16.34 | 18.34 |
| 80 | 5000 | 6.66 | 6.16 | 14.86 | 15.47 | 1.52 | 1.88 | 10.47 | 11.17 |
| 80 | 10000 | 6.20 | 6.12 | 15.79 | 16.18 | 3.46 | 4.09 | 16.99 | 18.24 |
| 80 | 15000 | 7.87 | 7.25 | 23.34 | 22.56 | 7.08 | 6.73 | 48.09 | 47.08 |
| 80 | 20000 | 8.91 | 9.49 | 28.82 | 26.87 | 9.29 | 9.75 | 54.16 | 35.07 |
| 80 | 22500 | 10.32 | 9.50 | 39.90 | 43.69 | 17.26 | 13.38 | 128.29 | 53.15 |
| 80 | 25000 | 12.43 | 14.85 | 98.46 | 74.38 | 12.06 | 13.58 | 44.65 | 67.25 |

**Table C1.** System Resource Usage Results of the Least Selective Query

| SC Result Filtering (%) | Number of Monitored Queries | Avg Memory Usage (GB) | | Max Memory Usage (GB) | | Avg CPU Usage (Threads) | | Max CPU Usage (Threads) | |
|---|---|---|---|---|---|---|---|---|---|
| | | Node-1 | Node-2 | Node-1 | Node-2 | Node-1 | Node-2 | Node-1 | Node-2 |
| - | 100 | 8.62 | 7.90 | 13.15 | 13.74 | 1.36 | 1.60 | 2.84 | 3.94 |
| - | 200 | 13.99 | 14.28 | 19.78 | 20.38 | 1.72 | 1.69 | 7.02 | 3.79 |
| - | 300 | 13.16 | 13.30 | 23.01 | 21.19 | 4.55 | 4.15 | 11.91 | 8.62 |

**Table C2.** System Resource Usage Results of the Low Selective Query

| SC Result Filtering (%) | Number of Monitored Queries | Avg Memory Usage (GB) | | Max Memory Usage (GB) | | Avg CPU Usage (Threads) | | Max CPU Usage (Threads) | |
|---|---|---|---|---|---|---|---|---|---|
| | | Node-1 | Node-2 | Node-1 | Node-2 | Node-1 | Node-2 | Node-1 | Node-2 |
| 0 | 100 | 9.83 | 9.36 | 14.76 | 15.05 | 1.60 | 1.70 | 3.34 | 3.91 |
| 0 | 200 | 15.02 | 14.16 | 21.90 | 21.00 | 3.18 | 3.09 | 6.68 | 6.88 |
| 0 | 300 | 14.75 | 15.44 | 22.99 | 22.37 | 5.14 | 2.73 | 13.26 | 5.39 |
| 20 | 100 | 8.13 | 7.12 | 12.61 | 12.61 | 1.69 | 1.75 | 4.09 | 3.15 |
| 20 | 200 | 11.19 | 11.71 | 18.10 | 17.83 | 2.47 | 2.73 | 6.15 | 6.92 |
| 20 | 300 | 15.47 | 14.76 | 23.41 | 21.50 | 3.99 | 3.65 | 10.18 | 6.95 |
| 20 | 400 | 12.95 | 13.51 | 22.47 | 23.01 | 4.40 | 4.45 | 9.48 | 8.24 |
| 40 | 100 | 6.78 | 6.62 | 12.24 | 0.01 | 1.44 | 1.33 | 3.63 | 2.80 |
| 40 | 200 | 10.22 | 10.56 | 15.64 | 16.46 | 1.83 | 2.45 | 4.35 | 6.14 |
| 40 | 300 | 13.04 | 12.63 | 18.67 | 19.35 | 2.42 | 3.37 | 5.74 | 7.77 |
| 40 | 400 | 15.59 | 14.95 | 22.38 | 22.85 | 3.82 | 3.88 | 10.76 | 7.73 |
| 40 | 500 | 16.14 | 16.19 | 25.42 | 23.36 | 3.82 | 4.48 | 12.16 | 10.00 |
| 60 | 100 | 3.29 | 3.07 | 5.09 | 5.03 | 1.05 | 1.00 | 1.85 | 1.90 |
| 60 | 200 | 7.90 | 8.36 | 14.40 | 13.60 | 1.31 | 1.37 | 4.10 | 3.79 |
| 60 | 300 | 9.58 | 10.19 | 16.57 | 16.12 | 1.79 | 1.72 | 5.07 | 6.24 |
| 60 | 400 | 11.07 | 11.97 | 18.06 | 18.21 | 2.51 | 3.70 | 6.69 | 9.65 |
| 60 | 500 | 12.79 | 13.81 | 20.78 | 19.93 | 3.25 | 4.32 | 9.34 | 10.76 |
| 60 | 750 | 15.64 | 15.48 | 25.72 | 24.78 | 5.61 | 4.19 | 19.62 | 7.39 |
| 80 | 500 | 8.98 | 9.11 | 15.08 | 14.67 | 2.12 | 2.22 | 5.85 | 6.38 |
| 80 | 750 | 11.26 | 11.25 | 17.61 | 17.73 | 3.65 | 3.80 | 9.93 | 8.22 |
| 80 | 1000 | 13.28 | 14.16 | 21.32 | 20.99 | 4.07 | 4.39 | 11.64 | 9.25 |
| 80 | 1250 | 16.30 | 14.70 | 25.10 | 23.37 | 4.60 | 4.59 | 11.73 | 11.09 |
| 80 | 1500 | 15.85 | 15.12 | 23.90 | 23.51 | 4.28 | 4.23 | 12.40 | 9.00 |

**Table C3.** System Resource Usage Results of the Middle Selective Query

| SC Result Filtering (%) | Number of Monitored Queries | Avg Memory Usage (GB) | | Max Memory Usage (GB) | | Avg CPU Usage (Threads) | | Max CPU Usage (Threads) | |
|---|---|---|---|---|---|---|---|---|---|
| | | **Node-1** | **Node-2** | **Node-1** | **Node-2** | **Node-1** | **Node-2** | **Node-1** | **Node-2** |
| 0 | 100 | 6.45 | 7.58 | 8.92 | 10.07 | 1.49 | 1.43 | 5.95 | 6.19 |
| 0 | 200 | 8.53 | 10.91 | 11.34 | 13.45 | 1.85 | 2.58 | 6.91 | 12.48 |
| 0 | 300 | 0.01 | 0.01 | 0.02 | 0.02 | 2.64 | 3.65 | 6.47 | 9.49 |
| 0 | 400 | 14.84 | 14.61 | 21.75 | 21.93 | 3.70 | 3.46 | 11.58 | 10.82 |
| 0 | 500 | 0.02 | 0.02 | 0.02 | 0.02 | 5.29 | 4.79 | 14.19 | 12.85 |
| 20 | 100 | 3.20 | 7.93 | 0.01 | 0.01 | 1.07 | 1.32 | 2.72 | 6.19 |
| 20 | 200 | 8.22 | 8.44 | 0.01 | 0.01 | 1.50 | 1.62 | 4.22 | 5.10 |
| 20 | 300 | 10.81 | 11.95 | 0.02 | 0.02 | 2.08 | 2.70 | 6.47 | 8.86 |
| 20 | 400 | 12.74 | 13.12 | 17.34 | 18.29 | 3.24 | 3.11 | 11.39 | 10.08 |
| 20 | 500 | 15.51 | 13.69 | 19.17 | 19.83 | 3.09 | 3.77 | 10.33 | 9.02 |
| 20 | 750 | 15.45 | 11.05 | 24.70 | 22.95 | 4.47 | 6.04 | 12.80 | 12.79 |
| 40 | 100 | 6.87 | 4.76 | 11.04 | 9.21 | 1.16 | 1.05 | 4.42 | 3.59 |
| 40 | 200 | 7.66 | 7.90 | 11.04 | 12.73 | 1.88 | 1.72 | 5.62 | 4.98 |
| 40 | 300 | 9.13 | 9.44 | 14.05 | 12.79 | 1.97 | 2.01 | 6.06 | 7.17 |
| 40 | 400 | 11.81 | 10.14 | 14.83 | 16.50 | 2.65 | 2.64 | 9.80 | 10.77 |
| 40 | 500 | 12.17 | 11.31 | 16.96 | 17.81 | 2.84 | 3.15 | 7.96 | 9.61 |
| 40 | 750 | 15.31 | 15.12 | 21.43 | 21.59 | 3.92 | 4.24 | 12.03 | 14.74 |
| 40 | 1000 | 13.25 | 14.31 | 23.38 | 23.28 | 6.24 | 4.80 | 16.51 | 11.85 |
| 60 | 250 | 8.14 | 6.71 | 10.26 | 9.14 | 1.58 | 1.52 | 7.23 | 7.15 |
| 60 | 500 | 9.78 | 9.40 | 14.90 | 16.73 | 2.49 | 2.49 | 7.78 | 8.54 |
| 60 | 750 | 12.35 | 11.07 | 17.59 | 14.69 | 3.30 | 3.50 | 11.26 | 9.98 |
| 60 | 1000 | 14.33 | 15.63 | 22.36 | 23.12 | 2.91 | 4.11 | 8.02 | 8.95 |
| 60 | 1250 | 15.31 | 15.20 | 23.48 | 24.29 | 3.29 | 5.69 | 6.74 | 20.86 |
| 80 | 500 | 6.61 | 6.54 | 10.98 | 9.11 | 1.42 | 1.63 | 9.18 | 10.45 |
| 80 | 750 | 8.03 | 8.87 | 11.92 | 10.65 | 1.73 | 1.85 | 10.42 | 13.18 |
| 80 | 1000 | 8.75 | 10.71 | 15.08 | 14.67 | 2.45 | 3.43 | 13.51 | 15.29 |
| 80 | 1500 | 11.90 | 13.62 | 16.06 | 17.71 | 3.33 | 3.57 | 18.13 | 19.39 |
| 80 | 2000 | 14.84 | 14.76 | 23.58 | 21.96 | 3.95 | 5.37 | 10.94 | 15.94 |
| 80 | 2500 | 17.54 | 17.98 | 26.88 | 25.47 | 4.65 | 5.02 | 8.98 | 12.60 |

**Table C4.** System Resource Usage Results of the High Selective Query

| SC Result Filtering (%) | Number of Monitored Queries | Avg Memory Usage (GB) | | Max Memory Usage (GB) | | Avg CPU Usage (Threads) | | Max CPU Usage (Threads) | |
|---|---|---|---|---|---|---|---|---|---|
| | | **Node-1** | **Node-2** | **Node-1** | **Node-2** | **Node-1** | **Node-2** | **Node-1** | **Node-2** |
| 0 | 500 | 7.91 | 8.54 | 11.38 | 11.05 | 1.97 | 1.82 | 12.01 | 11.98 |
| 0 | 1000 | 9.39 | 10.26 | 13.77 | 13.63 | 2.98 | 2.83 | 15.55 | 16.25 |
| 0 | 1500 | 11.81 | 12.74 | 18.09 | 17.97 | 4.08 | 4.54 | 13.19 | 18.60 |
| 0 | 2000 | 13.95 | 14.42 | 20.44 | 22.81 | 4.53 | 6.25 | 13.59 | 25.47 |
| 0 | 2500 | 17.87 | 18.05 | 25.09 | 26.19 | 3.62 | 6.33 | 14.18 | 18.91 |
| 20 | 500 | 8.48 | 8.14 | 11.55 | 11.42 | 1.45 | 1.63 | 9.14 | 10.78 |
| 20 | 1000 | 9.25 | 8.54 | 12.46 | 13.32 | 2.28 | 2.66 | 16.18 | 14.96 |
| 20 | 1500 | 11.85 | 11.15 | 15.93 | 14.68 | 3.96 | 4.60 | 19.53 | 23.49 |
| 20 | 2000 | 12.73 | 13.28 | 18.84 | 19.66 | 4.61 | 5.06 | 17.27 | 21.92 |
| 20 | 2500 | 15.15 | 15.90 | 0.02 | 0.02 | 5.15 | 6.93 | 16.85 | 23.13 |
| 20 | 3000 | 17.51 | 17.05 | 24.64 | 25.76 | 5.27 | 6.76 | 11.32 | 17.82 |
| 40 | 500 | 5.04 | 6.89 | 8.54 | 9.89 | 1.55 | 1.19 | 10.82 | 8.14 |
| 40 | 1000 | 8.49 | 8.76 | 12.55 | 12.37 | 2.14 | 2.07 | 12.25 | 11.83 |
| 40 | 2000 | 11.40 | 10.53 | 16.14 | 15.36 | 3.96 | 4.12 | 17.90 | 22.82 |
| 40 | 3000 | 14.97 | 14.02 | 21.27 | 19.16 | 5.78 | 5.98 | 22.18 | 22.84 |
| 40 | 4000 | 18.92 | 18.35 | 26.73 | 25.43 | 8.18 | 8.62 | 29.43 | 25.31 |
| 40 | 4500 | 16.33 | 15.79 | 24.93 | 24.05 | 8.44 | 7.38 | 21.74 | 21.22 |
| 60 | 500 | 6.33 | 6.41 | 8.83 | 10.81 | 1.18 | 1.44 | 6.33 | 8.79 |
| 60 | 1000 | 8.29 | 6.57 | 11.09 | 9.49 | 2.07 | 2.19 | 15.83 | 16.35 |
| 60 | 2000 | 10.28 | 11.05 | 15.01 | 14.62 | 3.21 | 3.33 | 15.35 | 17.77 |
| 60 | 3000 | 12.06 | 11.83 | 19.24 | 18.01 | 4.37 | 4.67 | 16.33 | 22.35 |
| 60 | 4000 | 13.37 | 16.04 | 21.14 | 21.53 | 6.33 | 6.88 | 19.05 | 20.29 |
| 60 | 5000 | 17.26 | 17.21 | 24.09 | 24.75 | 7.71 | 8.34 | 21.80 | 25.10 |
| 60 | 6000 | 17.14 | 19.43 | 26.04 | 25.96 | 7.72 | 6.98 | 19.27 | 17.25 |
| 80 | 500 | 6.48 | 6.97 | 8.82 | 9.83 | 1.20 | 1.16 | 7.22 | 5.83 |
| 80 | 1000 | 5.16 | 5.90 | 10.28 | 10.41 | 1.36 | 1.60 | 7.47 | 8.08 |
| 80 | 2500 | 7.29 | 5.89 | 13.07 | 12.66 | 2.37 | 2.48 | 8.48 | 9.46 |
| 80 | 5000 | 10.17 | 10.14 | 16.43 | 16.64 | 3.85 | 4.26 | 13.79 | 12.06 |
| 80 | 7500 | 12.68 | 12.90 | 19.18 | 19.83 | 4.73 | 5.52 | 11.93 | 11.71 |
| 80 | 10000 | 15.19 | 15.73 | 22.80 | 22.87 | 6.36 | 7.25 | 14.13 | 14.81 |
| 80 | 12500 | 17.57 | 17.87 | 26.76 | 27.59 | 9.57 | 10.77 | 21.32 | 22.98 |

**Table C5.** System Resource Usage Results of the Most Selective Query

| SC Result Filtering (%) | Number of Monitored Queries | Avg Memory Usage (GB) | | Max Memory Usage (GB) | | Avg CPU Usage (Threads) | | Max CPU Usage (Threads) | |
|---|---|---|---|---|---|---|---|---|---|
| | | **Node-1** | **Node-2** | **Node-1** | **Node-2** | **Node-1** | **Node-2** | **Node-1** | **Node-2** |
| 0 | 500 | 6.10 | 5.60 | 8.56 | 7.44 | 1.24 | 1.28 | 5.61 | 8.23 |
| 0 | 1000 | 7.81 | 7.11 | 11.53 | 11.08 | 1.96 | 2.18 | 11.55 | 12.47 |
| 0 | 2000 | 10.54 | 10.10 | 16.66 | 15.90 | 2.27 | 2.90 | 7.20 | 9.64 |
| 0 | 3000 | 12.62 | 12.98 | 19.61 | 19.10 | 4.65 | 5.66 | 10.79 | 14.68 |
| 0 | 4000 | 0.02 | 0.02 | 0.02 | 0.02 | 4.54 | 5.34 | 13.31 | 14.45 |
| 0 | 5000 | 18.29 | 17.04 | 27.15 | 25.72 | 7.94 | 7.65 | 26.16 | 17.39 |
| 20 | 500 | 4.87 | 6.93 | 7.51 | 11.04 | 1.22 | 1.01 | 7.35 | 3.55 |
| 20 | 1000 | 8.58 | 6.70 | 10.69 | 10.16 | 1.54 | 2.09 | 10.69 | 13.52 |
| 20 | 2000 | 10.23 | 9.70 | 12.43 | 14.74 | 3.13 | 3.89 | 19.15 | 19.12 |
| 20 | 3000 | 12.30 | 10.89 | 16.73 | 16.23 | 4.44 | 4.90 | 17.67 | 26.84 |
| 20 | 4000 | 14.23 | 12.92 | 19.36 | 17.54 | 5.90 | 6.85 | 22.11 | 22.90 |
| 20 | 5000 | 16.73 | 15.63 | 24.07 | 21.71 | 5.73 | 6.32 | 18.53 | 24.98 |
| 20 | 6000 | 17.54 | 17.57 | 26.18 | 26.65 | 7.04 | 8.03 | 17.05 | 18.26 |
| 40 | 500 | 7.49 | 5.70 | 10.55 | 10.51 | 1.20 | 1.26 | 6.66 | 11.51 |
| 40 | 1000 | 6.65 | 8.43 | 10.23 | 11.14 | 1.62 | 1.62 | 14.67 | 14.38 |
| 40 | 2000 | 8.84 | 8.94 | 14.17 | 13.38 | 2.66 | 2.81 | 16.11 | 18.24 |
| 40 | 3000 | 10.51 | 10.15 | 14.46 | 15.91 | 3.78 | 4.51 | 16.55 | 17.03 |
| 40 | 4000 | 13.15 | 12.75 | 19.02 | 18.97 | 4.34 | 6.02 | 17.04 | 23.26 |
| 40 | 5000 | 15.13 | 15.23 | 22.21 | 21.06 | 6.10 | 6.97 | 21.69 | 22.48 |
| 40 | 6000 | 17.89 | 18.25 | 26.06 | 25.99 | 6.78 | 7.34 | 15.22 | 19.23 |
| 40 | 7000 | 16.99 | 17.65 | 26.25 | 25.63 | 7.29 | 8.93 | 13.96 | 25.09 |
| 60 | 500 | 4.32 | 4.88 | 8.42 | 7.64 | 1.15 | 1.34 | 7.05 | 6.07 |
| 60 | 1000 | 5.42 | 6.47 | 9.48 | 9.37 | 1.50 | 1.72 | 9.07 | 9.76 |
| 60 | 2000 | 8.79 | 8.19 | 11.84 | 12.48 | 2.07 | 2.31 | 13.73 | 13.55 |
| 60 | 3000 | 8.37 | 10.08 | 13.88 | 14.19 | 3.20 | 3.51 | 14.65 | 19.09 |
| 60 | 4000 | 11.55 | 11.37 | 17.52 | 17.41 | 4.09 | 3.43 | 10.75 | 9.07 |
| 60 | 5000 | 14.72 | 13.75 | 22.52 | 21.52 | 4.66 | 5.18 | 11.90 | 16.38 |
| 60 | 6000 | 15.23 | 14.98 | 22.09 | 21.12 | 6.01 | 7.12 | 15.15 | 18.16 |
| 60 | 7000 | 15.45 | 15.21 | 23.61 | 22.75 | 6.85 | 6.27 | 15.85 | 16.21 |
| 60 | 8000 | 16.73 | 16.37 | 25.31 | 24.51 | 7.48 | 8.59 | 17.09 | 17.86 |
| 60 | 9000 | 16.83 | 16.69 | 27.99 | 24.01 | 8.81 | 7.75 | 28.91 | 16.99 |
| 80 | 500 | 6.62 | 6.60 | 9.00 | 10.32 | 1.06 | 1.12 | 5.39 | 6.50 |
| 80 | 1000 | 6.50 | 5.71 | 10.63 | 10.34 | 1.22 | 1.38 | 5.36 | 9.28 |
| 80 | 2500 | 7.96 | 7.09 | 11.28 | 11.35 | 1.90 | 1.71 | 9.65 | 6.61 |
| 80 | 5000 | 7.99 | 6.91 | 13.33 | 13.66 | 3.20 | 3.51 | 14.65 | 19.09 |
| 80 | 10000 | 10.56 | 10.09 | 16.93 | 16.81 | 5.54 | 6.72 | 15.22 | 13.36 |
| 80 | 15000 | 11.83 | 11.55 | 20.08 | 18.55 | 7.91 | 8.55 | 19.12 | 23.58 |
| 80 | 20000 | 14.57 | 14.54 | 22.87 | 25.26 | 9.40 | 10.19 | 21.63 | 23.81 |
| 80 | 22500 | 15.19 | 14.27 | 26.21 | 24.87 | 9.52 | 9.52 | 30.49 | 22.49 |
| 80 | 25000 | 13.38 | 13.96 | 24.96 | 25.89 | 8.88 | 11.25 | 22.78 | 28.39 |

## D Network Cost Tables

**Table D1.** Network Cost Results of the Least Selective Query

| SC Result Filtering (%) | Number of Monitored Queries | Query Count per Minute | Max Message Size per Query (MB) | Total Message Size per Query (MB) | Total Message Size per Second (MB) | Total Message Size (GB) | Query Count per Second |
|---|---|---|---|---|---|---|---|
| - | 100 | 10 | 21.46 | 81.49 | 13.58 | 7.96 | 0.17 |
| - | 200 | 20 | 21.46 | 81.49 | 27.17 | 15.92 | 0.33 |
| - | 300 | 30 | 21.46 | 81.49 | 40.75 | 23.88 | 0.50 |

**Table D2.** Network Cost Results of the Low Selective Query

| SC Result Filtering (%) | Number of Monitored Queries | Query Count per Minute | Max Message Size per Query (MB) | Total Message Size per Query (MB) | Total Message Size per Second (MB) | Total Message Size (GB) | Query Count per Second |
|---|---|---|---|---|---|---|---|
| 0 | 100 | 10 | 24.29 | 81.90 | 13.65 | 8.00 | 0.17 |
| 0 | 200 | 20 | 24.29 | 81.90 | 27.30 | 16.00 | 0.33 |
| 0 | 300 | 30 | 24.29 | 81.90 | 40.95 | 23.99 | 0.50 |
| 20 | 100 | 10 | 16.67 | 59.35 | 9.89 | 5.80 | 0.17 |
| 20 | 200 | 20 | 16.67 | 59.35 | 19.78 | 11.59 | 0.33 |
| 20 | 300 | 30 | 16.67 | 59.35 | 29.68 | 17.39 | 0.50 |
| 20 | 400 | 40 | 16.67 | 59.35 | 39.57 | 23.28 | 0.67 |
| 40 | 100 | 10 | 12.68 | 44.87 | 7.48 | 4.38 | 0.17 |
| 40 | 200 | 20 | 12.68 | 44.87 | 14.96 | 8.76 | 0.33 |
| 40 | 300 | 30 | 12.68 | 44.87 | 22.44 | 13.15 | 0.50 |
| 40 | 400 | 40 | 12.68 | 44.87 | 29.92 | 17.53 | 0.67 |
| 40 | 500 | 50 | 12.68 | 44.87 | 37.39 | 21.91 | 0.83 |
| 60 | 100 | 10 | 8.34 | 29.66 | 4.94 | 2.90 | 0.17 |
| 60 | 200 | 20 | 8.34 | 29.66 | 9.89 | 5.79 | 0.33 |
| 60 | 300 | 30 | 8.34 | 29.66 | 14.83 | 8.69 | 0.50 |
| 60 | 400 | 40 | 8.34 | 29.66 | 19.77 | 11.59 | 0.67 |
| 60 | 500 | 50 | 8.34 | 29.66 | 24.71 | 14.48 | 0.83 |
| 60 | 750 | 75 | 8.34 | 29.66 | 37.07 | 21.72 | 1.25 |
| 80 | 500 | 50 | 4.23 | 14.99 | 12.49 | 7.32 | 0.83 |
| 80 | 750 | 75 | 4.23 | 14.99 | 18.74 | 10.98 | 1.25 |
| 80 | 1000 | 100 | 4.23 | 14.99 | 24.99 | 14.64 | 1.67 |
| 80 | 1250 | 125 | 4.23 | 14.99 | 31.23 | 18.30 | 2.08 |
| 80 | 1500 | 150 | 4.23 | 14.99 | 37.48 | 21.96 | 2.50 |

**Table D3.** Network Cost Results of the Middle Selective Query

| SC Result Filtering (%) | Number of Monitored Queries | Query Count per Minute | Max Message Size per Query (MB) | Total Message Size per Query (MB) | Total Message Size per Second (MB) | Total Message Size (GB) | Query Count per Second |
|---|---|---|---|---|---|---|---|
| 0 | 100 | 100 | 10.73 | 40.43 | 67.39 | 3.95 | 1.67 |
| 0 | 200 | 200 | 10.73 | 40.43 | 134.78 | 7.90 | 3.33 |
| 0 | 300 | 60 | 10.73 | 40.43 | 40.43 | 11.85 | 1.00 |
| 0 | 400 | 80 | 10.73 | 40.43 | 53.91 | 15.79 | 1.33 |
| 0 | 500 | 100 | 10.73 | 40.43 | 67.39 | 19.74 | 1.67 |
| 20 | 100 | 50 | 8.61 | 30.21 | 25.18 | 2.95 | 0.83 |
| 20 | 200 | 50 | 8.61 | 30.21 | 25.18 | 5.90 | 0.83 |
| 20 | 300 | 75 | 8.61 | 30.21 | 37.76 | 8.85 | 1.25 |
| 20 | 400 | 100 | 8.61 | 30.21 | 50.35 | 11.80 | 1.67 |
| 20 | 500 | 100 | 8.61 | 30.21 | 50.35 | 14.75 | 1.67 |
| 20 | 750 | 75 | 8.61 | 30.21 | 37.76 | 22.13 | 1.25 |
| 40 | 100 | 50 | 6.55 | 22.99 | 19.16 | 2.25 | 0.83 |
| 40 | 200 | 50 | 6.55 | 22.99 | 19.16 | 4.49 | 0.83 |
| 40 | 300 | 75 | 6.55 | 22.99 | 28.74 | 6.74 | 1.25 |
| 40 | 400 | 100 | 6.55 | 22.99 | 38.32 | 8.98 | 1.67 |
| 40 | 500 | 100 | 6.55 | 22.99 | 38.32 | 11.23 | 1.67 |
| 40 | 750 | 150 | 6.55 | 22.99 | 57.48 | 16.84 | 2.50 |
| 40 | 1000 | 100 | 6.55 | 22.99 | 38.32 | 22.45 | 1.67 |
| 60 | 250 | 125 | 4.31 | 15.14 | 31.55 | 3.70 | 2.08 |
| 60 | 500 | 100 | 4.31 | 15.14 | 25.24 | 7.39 | 1.67 |
| 60 | 750 | 150 | 4.31 | 15.14 | 37.86 | 11.09 | 2.50 |
| 60 | 1000 | 100 | 4.31 | 15.14 | 25.24 | 14.79 | 1.67 |
| 60 | 1250 | 125 | 4.31 | 15.14 | 31.55 | 18.49 | 2.08 |
| 80 | 500 | 500 | 2.18 | 7.68 | 64.02 | 3.75 | 8.33 |
| 80 | 750 | 750 | 2.18 | 7.68 | 96.03 | 5.63 | 12.50 |
| 80 | 1000 | 500 | 2.18 | 7.68 | 64.02 | 7.50 | 8.33 |
| 80 | 1500 | 750 | 2.18 | 7.68 | 96.03 | 11.25 | 12.50 |
| 80 | 2000 | 250 | 2.18 | 7.68 | 32.01 | 15.00 | 4.17 |
| 80 | 2500 | 250 | 2.18 | 7.68 | 32.01 | 18.76 | 4.17 |

**Table D4.** Network Cost Results of the High Selective Query

| SC Result Filtering (%) | Number of Monitored Queries | Query Count per Minute | Max Message Size per Query (MB) | Total Message Size per Query (MB) | Total Message Size per Second (MB) | Total Message Size (GB) | Query Count per Second |
|---|---|---|---|---|---|---|---|
| 0 | 500 | 500 | 2.32 | 8.16 | 67.98 | 3.98 | 8.33 |
| 0 | 1000 | 500 | 2.32 | 8.16 | 67.98 | 7.97 | 8.33 |
| 0 | 1500 | 300 | 2.32 | 8.16 | 40.79 | 11.95 | 5.00 |
| 0 | 2000 | 400 | 2.32 | 8.16 | 54.39 | 15.93 | 6.67 |
| 0 | 2500 | 250 | 2.32 | 8.16 | 33.99 | 19.92 | 4.17 |
| 20 | 500 | 500 | 1.80 | 6.56 | 54.67 | 3.20 | 8.33 |
| 20 | 1000 | 500 | 1.80 | 6.56 | 54.67 | 6.41 | 8.33 |
| 20 | 1500 | 600 | 1.80 | 6.56 | 65.61 | 9.61 | 10.00 |
| 20 | 2000 | 500 | 1.80 | 6.56 | 54.67 | 12.81 | 8.33 |
| 20 | 2500 | 500 | 1.80 | 6.56 | 54.67 | 16.02 | 8.33 |
| 20 | 3000 | 300 | 1.80 | 6.56 | 32.80 | 19.22 | 5.00 |
| 40 | 500 | 500 | 1.28 | 4.97 | 41.38 | 2.42 | 8.33 |
| 40 | 1000 | 500 | 1.28 | 4.97 | 41.38 | 4.85 | 8.33 |
| 40 | 2000 | 1000 | 1.28 | 4.97 | 82.77 | 9.70 | 16.67 |
| 40 | 3000 | 750 | 1.28 | 4.97 | 62.07 | 14.55 | 12.50 |
| 40 | 4000 | 1000 | 1.28 | 4.97 | 82.77 | 19.40 | 16.67 |
| 40 | 4500 | 900 | 1.28 | 4.97 | 74.49 | 21.82 | 15.00 |
| 60 | 500 | 500 | 0.96 | 4.00 | 33.33 | 1.95 | 8.33 |
| 60 | 1000 | 1000 | 0.96 | 4.00 | 66.66 | 3.91 | 16.67 |
| 60 | 2000 | 1000 | 0.96 | 4.00 | 66.66 | 7.81 | 16.67 |
| 60 | 3000 | 1000 | 0.96 | 4.00 | 66.66 | 11.72 | 16.67 |
| 60 | 4000 | 1000 | 0.96 | 4.00 | 66.66 | 15.62 | 16.67 |
| 60 | 5000 | 1000 | 0.96 | 4.00 | 66.66 | 19.53 | 16.67 |
| 60 | 6000 | 1000 | 0.96 | 4.00 | 66.66 | 23.44 | 16.67 |
| 80 | 500 | 500 | 0.48 | 1.70 | 14.13 | 0.83 | 8.33 |
| 80 | 1000 | 500 | 0.48 | 1.70 | 14.13 | 1.66 | 8.33 |
| 80 | 2500 | 500 | 0.48 | 1.70 | 14.13 | 4.14 | 8.33 |
| 80 | 5000 | 500 | 0.48 | 1.70 | 14.13 | 8.28 | 8.33 |
| 80 | 7500 | 750 | 0.48 | 1.70 | 21.20 | 12.42 | 12.50 |
| 80 | 10000 | 1000 | 0.48 | 1.70 | 28.27 | 16.56 | 16.67 |
| 80 | 12500 | 1000 | 0.48 | 1.70 | 28.27 | 20.70 | 16.67 |

**Table D5.** Network Cost Results of the Most Selective Query

| SC Result Filtering (%) | Number of Monitored Queries | Query Count per Minute | Max Message Size per Query (MB) | Total Message Size per Query (MB) | Total Message Size per Second (MB) | Total Message Size (GB) | Query Count per Second |
|---|---|---|---|---|---|---|---|
| 0 | 500 | 500 | 1.36 | 3.86 | 32.13 | 1.88 | 8.33 |
| 0 | 1000 | 500 | 1.36 | 3.86 | 32.13 | 3.77 | 8.33 |
| 0 | 2000 | 200 | 1.36 | 3.86 | 12.85 | 7.53 | 3.33 |
| 0 | 3000 | 300 | 1.36 | 3.86 | 19.28 | 11.30 | 5.00 |
| 0 | 4000 | 400 | 1.36 | 3.86 | 25.71 | 15.06 | 6.67 |
| 0 | 5000 | 500 | 1.36 | 3.86 | 32.13 | 18.83 | 8.33 |
| 20 | 500 | 500 | 1.06 | 3.25 | 27.06 | 1.59 | 8.33 |
| 20 | 1000 | 1000 | 1.06 | 3.25 | 54.12 | 3.17 | 16.67 |
| 20 | 2000 | 1000 | 1.06 | 3.25 | 54.12 | 6.34 | 16.67 |
| 20 | 3000 | 1000 | 1.06 | 3.25 | 54.12 | 9.51 | 16.67 |
| 20 | 4000 | 1000 | 1.06 | 3.25 | 54.12 | 12.68 | 16.67 |
| 20 | 5000 | 1000 | 1.06 | 3.25 | 54.12 | 15.86 | 16.67 |
| 20 | 6000 | 600 | 1.06 | 3.25 | 32.47 | 19.03 | 10.00 |
| 40 | 500 | 500 | 0.75 | 2.63 | 21.89 | 1.28 | 8.33 |
| 40 | 1000 | 1000 | 0.75 | 2.63 | 43.79 | 2.57 | 16.67 |
| 40 | 2000 | 1000 | 0.75 | 2.63 | 43.79 | 5.13 | 16.67 |
| 40 | 3000 | 750 | 0.75 | 2.63 | 32.84 | 7.70 | 12.50 |
| 40 | 4000 | 1000 | 0.75 | 2.63 | 43.79 | 10.26 | 16.67 |
| 40 | 5000 | 1000 | 0.75 | 2.63 | 43.79 | 12.83 | 16.67 |
| 40 | 6000 | 600 | 0.75 | 2.63 | 26.27 | 15.39 | 10.00 |
| 40 | 7000 | 700 | 0.75 | 2.63 | 30.65 | 17.96 | 11.67 |
| 60 | 500 | 500 | 0.56 | 2.25 | 18.76 | 1.10 | 8.33 |
| 60 | 1000 | 1000 | 0.56 | 2.25 | 37.52 | 2.20 | 16.67 |
| 60 | 2000 | 1000 | 0.56 | 2.25 | 37.52 | 4.40 | 16.67 |
| 60 | 3000 | 1000 | 0.56 | 2.25 | 37.52 | 6.60 | 16.67 |
| 60 | 4000 | 400 | 0.56 | 2.25 | 15.01 | 8.79 | 6.67 |
| 60 | 5000 | 500 | 0.56 | 2.25 | 18.76 | 10.99 | 8.33 |
| 60 | 6000 | 600 | 0.56 | 2.25 | 22.51 | 13.19 | 10.00 |
| 60 | 7000 | 700 | 0.56 | 2.25 | 26.26 | 15.39 | 11.67 |
| 60 | 8000 | 800 | 0.56 | 2.25 | 30.01 | 17.59 | 13.33 |
| 60 | 9000 | 900 | 0.56 | 2.25 | 33.77 | 19.79 | 15.00 |
| 80 | 500 | 500 | 0.28 | 0.81 | 6.79 | 0.40 | 8.33 |
| 80 | 1000 | 500 | 0.28 | 0.81 | 6.79 | 0.80 | 8.33 |
| 80 | 2500 | 1000 | 0.28 | 0.81 | 13.58 | 1.99 | 16.67 |
| 80 | 5000 | 1000 | 0.28 | 0.81 | 13.58 | 3.98 | 16.67 |
| 80 | 10000 | 1000 | 0.28 | 0.81 | 13.58 | 7.96 | 16.67 |
| 80 | 15000 | 1000 | 0.28 | 0.81 | 13.58 | 11.94 | 16.67 |
| 80 | 20000 | 1000 | 0.28 | 0.81 | 13.58 | 15.91 | 16.67 |
| 80 | 22500 | 1000 | 0.28 | 0.81 | 13.58 | 17.90 | 16.67 |
| 80 | 25000 | 1000 | 0.28 | 0.81 | 13.58 | 19.89 | 16.67 |
